# Long noncoding RNA FAM225B facilitates proliferation and metastasis of nasopharyngeal carcinoma cells by regulating miR-613/CCND2 axis

**DOI:** 10.17305/bjbms.2021.5691

**Published:** 2021-06-17

**Authors:** Weijun Dai, Yi Shi, Weiqi Hu, Chenjie Xu

**Affiliations:** 1 Department of Otolaryngology, Shanghai Pudong New Area Gongli Hospital, Shanghai, China; 2 Department of Otolaryngology, Minhang Hospital, Fudan University, Shanghai, China; 3 Department of Otolaryngology Head and Neck Surgery, Shanghai Ninth People’s Hospital, School of Medicine, Shanghai Jiaotong University, Shanghai, China

**Keywords:** Nasopharyngeal carcinoma, lncRNA, FAM225B, metastasis, CCND2

## Abstract

Growing evidence has suggested that abnormally expressed long non-coding RNAs (lncRNAs) play critical regulatory roles in nasopharyngeal carcinoma (NPC) pathogenesis. Family with sequence similarity 225 members B (FAM225B) is a novel lncRNA that has been implicated in several human cancers, yet its role in the context of NPC remains largely unclear. The aim of this study was to determine the expression level of FAM225B and its clinical significance in NPC patients. We observed a remarkable increase of FAM225B in NPC tissues and cell lines compared with controls. Furthermore, highly expressed FAM225B was closely correlated with advanced TNM (tumor, node metastases) stage, distant metastasis, and poor overall survival. Interestingly, loss-of-function analysis revealed that FAM225B knockdown significantly inhibited tumor growth *in vitro* and *in vivo*, and decreased the migratory and invasive capacity of NPC cells. Mechanically, FAM225B functioned as an endogenous sponge by competing for miR-613 binding to upregulate CCND2 expression. More importantly, rescue experiments further demonstrated that the suppressive impacts of FAM225B knockdown on cell proliferation, migration, and invasion were significantly reversed after CCND2 overexpression. Taken all together, these findings highlight FAM225B as an oncogene that promotes NPC proliferation and metastasis through miR-613/CCND2 axis.

## INTRODUCTION

Metastasis is a dominating obstacle to the successful treatment of solid tumors, including nasopharyngeal carcinoma (NPC), with a high incidence in southern China [1,2]. In 2020, there were 133,354 new NPC cases and 80,008 deaths worldwide [3]. The occurrence of NCP is closely related to Epstein-Barr virus (EBV) infection, and many signal pathways and dysregulated microRNAs (miRNAs) have been identified to be implicated during NPC progression [4,5]. Although radiochemotherapy has substantially improved the prognosis, approximately 20%-30% NPC cases still eventually develop locoregional recurrence or distant metastasis [6,7]. For this reason, understanding the potential mechanisms of NPC metastasis is crucial for the prognosis evaluation and cancer treatment.

Long non-coding RNAs (lncRNAs) are a group of transcribed RNA molecules with a length of over 200 nucleotides. Accumulating evidence demonstrated that dysregulated lncRNAs, such as DANCR, SMAD5-AS1, LINC00460, and ZFAS1, have been broadly implicated in the NPC development and progression [8-11]. Family with sequence similarity 225 members B (FAM225B), also known as LINC00256B, is a newly discovered lncRNA located on chromosome 9q32. Recently, several studies reported that FAM225B was abnormally expressed in renal cell carcinoma, bladder cancer, papillary thyroid carcinoma, and glioblastoma and can be used as a candidate prognostic biomarker [12-15]. A recent study demonstrated that knockdown of FAM225B significantly suppressed the proliferation of scar fibroblasts by regulating autophagy [16]. Nevertheless, the clinical significance and biological function of FAM225B in NPC tumorigenesis are still unknown.

CCND2 encodes cyclin D2, a protein belonging to the highly conserved cyclin family that functions as a regulator of CDK 4/6 in the cell cycle G1/S transition [17]. The previous studies indicated that CCND2 expression was frequently up-regulated and acted as an oncogene to enhance tumor growth, migration, invasion, stemness, and chemoresistance in a wide variety of human cancers [18-21]. In NPC, CCND2 was reported to be highly expressed and associated with T classification, clinical stage as well as worse outcome [22]. Furthermore, silencing of CCND2 could significantly inhibit cell growth and colony formation of NPC cells [23]. These findings demonstrated the significance of CCND2 in NPC pathogenesis. However, a more detailed role and mechanism behind CCND2 overexpression in NPC have not been elucidated.

In this study, we first determined the expression level of FAM225B and its clinical significance in NPC patients. We found that FAM225B expression was dramatically elevated, and patients with higher FAM225B had a worse outcome. Moreover, FAM225B knockdown significantly inhibited cell proliferation, migration and invasion. Mechanistically, FAM225B functioned as a miRNA sponge of miR-613 to up-regulate CCND2 expression. Overall, our results revealed a novel mechanism underlying NPC tumor growth and metastasis.

## MATERIALS AND METHODS

### Patient tissues and cell cultures

The study was approved by the Institutional Ethical Review Boards of Shanghai Ninth People’s Hospital and complied with the guidelines of the Declaration of Helsinki. A total of 56 NPC tissues and adjacent normal tissues with informed consent were collected by biopsy between July 2016 and June 2018. None of the patients had received any chemotherapy or radiotherapy before biopsy. NPC cell lines, including HONE-1, C666-1, CNE-1, CNE-2, and SUNE-1, and human immortalized nasopharyngeal epithelial cell line (NP69) were purchased from the Cell Bank of Shanghai Institutes for Biological Sciences, Chinese Academy of Sciences (Shanghai, China). Cells were incubated at 37°C and supplemented with 5% CO_2_ in a humidified incubator.

### Oligonucleotides and transfection

The synthetic miR-613 mimics, miR-613 inhibitor (miR-613-in), miRNA negative control (miRNA-NC), siRNA, and shRNA against FAM225B were purchased from GenePharma (Shanghai, China). The full-length of CCND2 was constructed into the pcDNA3.1 (Invitrogen). Lipofectamine 2000 (Invitrogen) was used for transfections with 50 nM oligonucleotides or 1 μg plasmids following the manufacturer’s protocol.

### Cell proliferation assays

The proliferative capacity of NPC cells was detected using CCK-8 assay and colony formation assay. For CCK-8 assay, cells were planted into 96-well plates at 5 × 10^3^ cells/well. Cell viability was measured every 24 hour by adding 10 μl CCK-8 solution into each well. The absorbance at 450 nm was detected with a microplate reader. For colony formation assay, cells (300 per well) were planted into 6-well plates for 14 days. The numbers of colonies containing more than 50 cells were stained with crystal violet and counted.

### RNA extraction and Quantitative reverse transcription polymerase chain reaction (qRT-PCR)

RNA extraction of NPC tissues and cultured cells was performed using Trizol reagent (Invitrogen). cDNA was reverse transcribed using a PrimeScript RT Master Mix (TaKaRa Bio, Shiga, Japan). qRT-PCR reactions were performed using the SYBR Premix Ex Taq II (TaKaRa) on an ABI 7500 Real-Time PCR system. The relative expression was calculated using the 2^−ΔΔCt^ method and normalized to U6 or β-actin. Primers were synthesized by Sangon Biotech (shanghai, China) as follows: FAM225B:5’-AAGGGTTAGATGTGGGTGGG-3’(forward) and 5’-CTCTCTAGTGACGCCCTCTG-3’(reverse);miR-613: 5’-GGAATGTTCCTTCTTTGC-3’(forward) and 5’-GAACATGTCTGCGTATCTC-3’(reverse); CCND2: 5’-GAGAAGCTGTCTCTGATCCGCA-3’(forward) and 5’-CTTCCAGTTGCGATCATCGACG-3’(reverse); U6 5’-CTCGCTTCGGCAGCACAT-3’(forward) and 5’-TTTGCGTGTCATCCTTGCG-3’(reverse);β-actin: 5’-CTTAGTTGCGTTACACCCTTTCTTG-3’(forward) and 5’-CTGTCACCTTCACCGTTCCAGTTT -3’ (reverse); β-actin.

### Western blot analysis

Protein lysates were extracted from cultured cells, separated by 10% SDS-PAGE and electrophoretically transferred to polyvinylidene difluoride membrane (Millipore). The membrane was incubated with primary antibodies against CCND2 (1:1000; ab230883), MMP9 (1:1000; ab76003), E-cadherin (1:1000; ab40772), N-cadherin (1:1000; ab76011), and Vimentin (1:1000; ab92547) overnight at 4°C and then incubated with secondary antibody for 1 hour at 37°C. Signals were visualized by enhanced chemiluminescence (Millipore, Bedford, MA).

### Transwell migration and invasion assays

Migration and invasion assays were performed using the Transwell chamber (BD Biosciences, Bedford, MA). For invasion assay, 5 × 10^4^ cells in serum-free medium were seeded into the upper chamber pre-coated with Matrigel (BD; without Matrigel for migration assay). Medium with 10% Fetal bovine serum was added to the lower chamber. After a 24-hour incubation, cells in the lower surface fixed and stained with crystal violet, and then counted under a microscope.

### Luciferase reporter assay

The wild type (wt) or mutant (mut) fragment of CCND2 3’ UTR and FAM225B containing the binding site of miR-613 was inserted into the pMIR-REPORT vector. NPC cells were co-transfected with the reporter vectors and the miR-613 mimics or miRNA negative control. Luciferase activity was measured 48 hour post-transfection using the Dual-Luciferase Reporter Assay System (Promega, Madison, WI, USA).

### Tumor xenograft assay

Cells (3.0 ×1 0^6^) stably expressing sh-FAM225B or sh-NC were subcutaneously injected into the posterior flank of 4-week-old female BALB/c nude mice (6 mice each group). The tumor volume was measured with a caliper weekly. After 6 weeks, the nude mice were euthanized and the tumors were collected and weighed. All animal experiments were performed in accordance with the Animal Care and Use guidelines of Shanghai Ninth People’s Hospital.

### Statistical analysis

Each experiment was performed at least three times. Statistical analysis was performed using SPSS 22.0 software (IBM, Armonk, NY, USA). Data were shown as the mean ± standard deviation. The associations between FAM225B and clinicopathological features were analyzed by Chi-square test. Correlations between FAM225B and miR-613 or CCND2 were analyzed by Pearson correlation test. Survival rate was evaluated by the Kaplan–Meier method and the log-rank test. *p* < 0.05 was considered statistically significant.

## RESULTS

FAM225B is frequently up-regulated and correlated with poor overall survival in NPC

**FIGURE 1:**
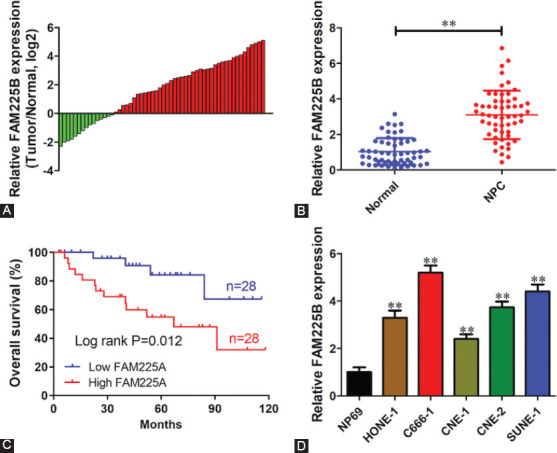
FAM225B is frequently upregulated and correlated with poor overall survival in NPC. (A,B) qRT-PCR was performed to determine FAM225B expression in 56 NPC tissues and adjacent normal tissues. (C) Kaplan–Meier analysis of overall survival according to the FAM225B expression. The high and low level was split referring to median value. (D) Relative expression level of FAM225B in NPC cell lines and NP69 cells. **p < 0.01. FAM225B: Family with sequence similarity 225 member B; NPC: Nasopharyngeal carcinoma; qRT-PCR: Quantitative reverse transcription polymerase chain reaction.

As shown in Figure 1A and 1B, the FAM225B expression in NPC samples was higher than that of their corresponding adjacent normal tissues. In addition, Kaplan–Meier survival analysis revealed that overexpressed FAM225B was significantly associated with unfavorable prognosis of patients with NPC (Figure 1C, *p* = 0.018). Further correlation analysis between FAM225B expression and clinical pathological feathers demonstrated that high FAM225B level was strongly related to TNM stage (*p* = 0.003), T stage (P=0.016), and distant metastasis (P=0.015), but not to age, sex, N stage, and EBV DNA of the patients (Table 1). Consistent with the results obtained in NPC tissues, FAM225B expression increased by approximately 2.3- to 5.2-fold in HONE-1, C666-1, CNE-1, CNE-2, and SUNE-1 cells compared with NP69 cells (Figure 1D). These results indicate that FAM225B may be a crucial regulator in NPC progression.

**TABLE 1:**
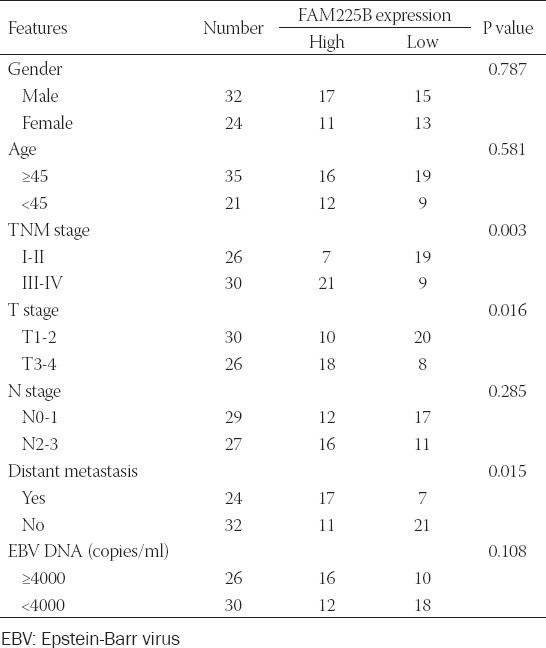
The correlation of FAM225B expression and clinical characteristics in NPC

### Knockdown of FAM225B inhibits the proliferation of NPC cells *in vitro* and *in vivo*

**FIGURE 2:**
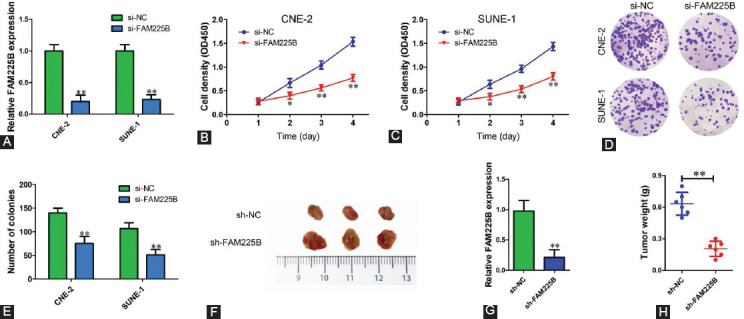
Knockdown of FAM225B inhibits the proliferation of NPC cells *in vitro* and *in vivo*. (A) Relative expression level of FAM225B in CNE-2 and SUNE-1 cells transfected with si-NC or si-FAM225B. (B,C) CCK-8 assay was performed to determine the effect of FAM225B knockdown on the viability of CNE-2 (B) and SUNE-1 (C) cells. (D,E) FAM225B knockdown inhibited cell proliferation detected using colony formation assay. (F-H) SUNE-1 cells stably expressing sh-FAM225B or sh-NC were subcutaneously injected into nude mice (*n*=6). Representative images of the tumors (F), relative FAM225B expression (G), and tumor weight (H) from each group. *p < 0.05, **p < 0.01. CCK-8: Cell counting kit-8; FAM225B: Family with sequence similarity 225 member B; NPC: Nasopharyngeal carcinoma; sh-FAM225B: Short hairpin RNA targeting FAM225B; sh-NC: Short hairpin RNA negative control; si-FAM225B: Small interfering RNA targeting FAM225B; si-NC: Small interfering

To evaluate the function of FAM225B in NPC, loss-of-function assays were performed in CNE-2 and SUNE-1 cells through specific siRNA for FAM225B. The knockdown of FAM225B expression was first validated using qRT-PCR (Figure 2A). CCK-8 assay results demonstrated that silence of FAM225B could dramatically repress the cell viability of CNE-2 and SUNE-1 cells, respectively, (Figure 2B and 2C). Colony formation assay showed that the clonogenicity of NPC cells was obviously reduced after FAM225B knockdown (Figure 2D and 2E). To further assess the effect of FAM225B on tumor growth *in vivo*, SUNE-1 cells stably expressing sh-FAM225B or sh-NC were subcutaneously injected into nude mice. The tumor weight from the FAM225B downregulation group was markedly decreased compared with the control group (Figure 2F and 2H). As expected, FAM225B expression of the sh-FAM225B group was significantly reduced (Figure 2G). These findings suggest that downregulation of FAM225B significantly suppresses the proliferation of NPC cells.

### Knockdown of FAM225B suppresses the migration and invasion of NPC cells *in vitro*

**FIGURE 3:**
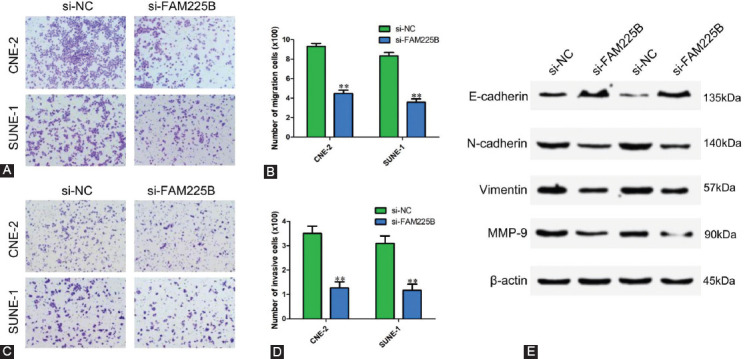
Knockdown of FAM225B suppresses the migration and invasion of NPC cells in vitro. (A,B) Transwell assay without Matrigel was used to detect migration of CNE-2 and SUNE-1 cells transfected with si-NC or si-FAM225B. (C,D) Transwell assay with Matrigel was used to detect invasion of CNE-2 and SUNE-1 cells transfected with si-NC or si-FAM225B. (E) Western blot assay was used to detect the expression of E-cadherin, N-cadherin, Vimentin and MMP-9 in CNE-2 and SUNE-1 cells transfected with si-NC or si-FAM225B. ** p < 0.01. FAM225B: Family with sequence similarity 225 member B; MMP-9: Matrix metallopeptidase 9; si-FAM225B: Small interfering RNA targeting FAM225B; si-NC: Small interfering RNA negative control.

As mentioned above, our clinical characteristics analysis indicates that FAM225B might also regulate the metastasis of NPC cells. Next, we determined whether FAM225B could affect the migratory and invasive capability of NPC cells using Transwell assay. As shown in Figure 3A and 3B, FAM225B knockdown significantly reduced the number of migratory cells compared with that of the si-NC group in both CNE-2 and SUNE-1 cells. Simultaneously, a Transwell assay with Matrigel showed that downregulation of FAM225B strongly inhibited the invasion of NPC cells (Figure 3C and 3D). Western blot was also performed to assess the effects of FAM225B knockdown on metastasis-related proteins. We observed that down-regulation of FAM225B significantly decreased the expression of N-cadherin, Vimentin and MMP-9, while increased E-cadherin expression (Figure 3E). Our data imply that FAM225B knockdown contributes to the inhibition of NPC metastasis.

### miR-613 is identified as downstream of FAM225B

**FIGURE 4:**
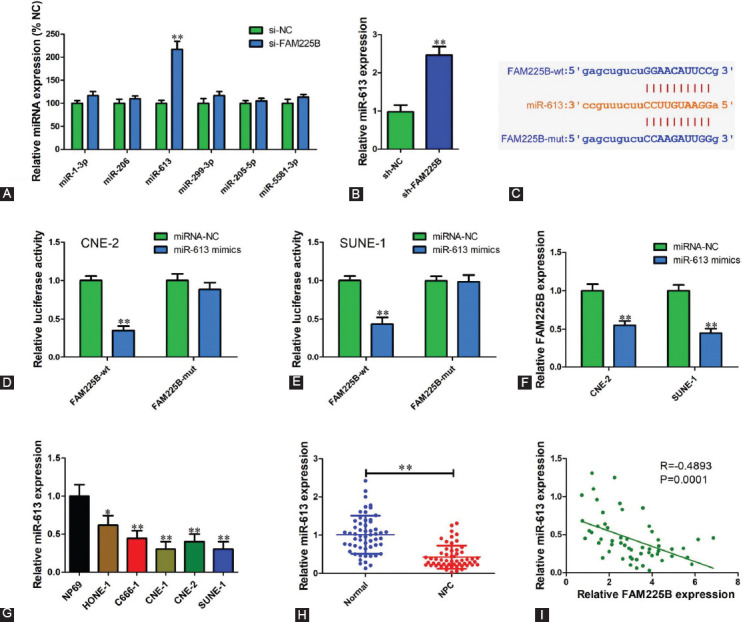
miR-613 is identified as downstream of FAM225B. (A) FAM225B was predicted to contain binding sites of miR-1-3p, miR-206, miR-613, miR-299-3p, miR-205-5p and miR-5581-3p using starBase. qRT-PCR was performed to determine their expression levels in SUNE-1 cells transfected with si-FAM225B or si-NC. (B) Relative miR-613 expression of sh-FAM225B and sh-NC group in xenograft tissues. (C) Wild type and mutant FAM225B sequence of miR-613 predicted using starBase. (D,E) FAM225B-wt or FAM225B-mut reporter vector was transfected into CNE-2 (D) and SUNE-1 (E) cells with miR-613 mimics or miRNA negative. Relative luciferase activity was measured using the Dual-Luciferase Reporter Assay System. (F) qRT-PCR was used to detect FAM225B expression level in CNE-2 and SUNE-1 cells transfected with miR-613 mimics or miRNA negative control. (G) Relative expression level of miR-613 in NPC cell lines and NP69 cells determined using qRT-PCR. (H) Relative expression level of miR-613 in NPC tissues and adjacent normal tissues determined using qRT-PCR. (I) Pearson correlation analysis of miR-613 and FAM225B in 56 NPC tissues. *p < 0.05, **p < 0.01. FAM225B: Family with sequence similarity 225 member B; miR: MicroRNA; miRNA-NC: MicroRNA negative control; mut: Mutant; NPC: Nasopharyngeal carcinoma; sh-FAM225B: Short hairpin RNA targeting FAM225B; sh-NC: Short hairpin RNA negative control; si-FAM225B: Small interfering RNA targeting FAM225B; si-NC: Small interfering RNA negative control; wt: Wild type; qRT-PCR: Quantitative reverse transcription polymerase chain reaction.

To illustrate the underlying mechanism by which FAM225B promotes NPC progression, bioinformatics analysis was performed using starBase. FAM225B was predicted to contain putative binding sites for six miRNAs (miR-1-3p, miR-206, miR-613, miR-299-3p, miR-205-5p, and miR-5581-3p). We found that miR-613 expression level was most significantly up-regulated when knockdown of FAM225B in SUNE-1 cells (Figure 4A). Besides, miR-613 expression of sh-FAM225B group was increased 2.48-fold compared with sh-NC group (Figure 4B). Next, we constructed wild type (wt) and mutant (mut) FAM225B reporter vectors that contain predicted binding sequence of miR-613 (Figure 4C). Luciferase reporter assay showed that miR-613 mimics significantly reduced the luciferase activity of wt-FAM225B, but not mut-FAM225B (Figure 4D and 4E). After transfection with miR-613 mimics, the FAM225B expression was dramatically reduced compared with miRNA negative control (Figure 4F). We also found a lower miR-613 level in both NPC cell lines and tissues (Figure 4G and 4H). In addition, there was a strong negative correlation between miR-613 and FAM225B in NPC tissues (Figure 4I, R = −0.4893, *p* = 0.0001). These results indicate that miR-613 is a downstream target of FAM225B.

### FAM225B up-regulates CCND2 expression through inhibiting miR-613

**FIGURE 5:**
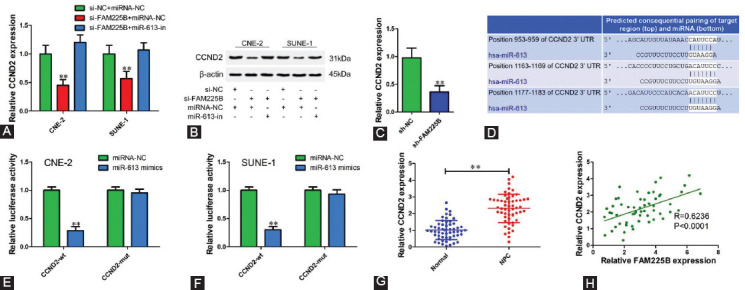
FAM225B up-regulates CCND2 expression through inhibiting miR-613. (A,B) Relative mRNA (A) and protein (B) expression of CCNB2 in CNE-2 and SUNE-1 cells transfected with si-NC or si-FAM225B and miRNA negative control or miR-613 inhibitor. (C) Relative CCND2 expression of sh-FAM225B and sh-NC group in xenograft tissues. (D) Predicted binding sites of miR-613 in CCND2 3'UTR using TargetScan. (E, F) Relative luciferase activity in CNE-2 (E) and SUNE-1 (F) cells transfected with CCND2-wt or CCND2-mut reporter vector with miR-613 mimics or miRNA negative. (G) Relative expression level of CCND2 in NPC tissues and adjacent normal tissues determined using qRT-PCR. (H) Pearson correlation analysis of CCDN2 and FAM225B in 56 NPC tissues. **p < 0.01. CCND2: Cyclin D2; FAM225B: Family with sequence similarity 225 member B; miR: MicroRNA; miRNA-NC: MicroRNA negative control; miR-613-in: microRNA-613 inhibitor; mut: Mutant; NPC: Nasopharyngeal carcinoma; sh-FAM225B: Short hairpin RNA targeting FAM225B; sh-NC: Short hairpin RNA negative control; si-FAM225B: Small interfering RNA targeting FAM225B; si-NC: Small interfering RNA negative control; wt: Wild type; qRT-PCR: Quantitative reverse transcription polymerase chain reaction.

Emerging evidence has shown that CCND2 is a well-known oncogene in tumors. To test whether FAM225B could regulate CCND2 expression via targeting miR-613, si-FAM225B and miR-613 inhibitors were co-transfected into CNE-2 and SUNE-1 cells. Compared with siRNA negative control, both mRNA and protein levels were decreased after transfection with si-FAM225B. However, miR-613 inhibitor reversed the inhibition of CCND2-induced by FAM225B knockdown (Figure 5A and 5B). Downregulated CCND2 expression was observed in sh-FAM225B xenograft tissues (Figure 5C). Online bioinformatics prediction revealed that the 3’UTR of CCND2 contains three binding sites of miR-613 (Figure 5D). Luciferase reporter assay results showed a decreased luciferase activity after co-transfection of miR-613 mimics and wt-CCND2 into NPC cells (Figure 5E and 5F). We also determined the CCND2 expression in NPC tissues and found that CCND2 expression in NPC tissues was up-regulated 2.3-fold compared with adjacent normal tissues (Figure 5G). Correlation analysis showed that CCND2 expression in NPC tissues was positively correlated with FAM225B (Figure 5H; R = 0.6236, *p* < 0.0001). Collectively, these observations suggest FAM225B functions as a competing endogenous RNA (ceRNA) to modulate CCND2 level by decoying miR-613.

### Overexpression of CCND2 reverses malignant phenotypes inhibition-induced by FAM225B knockdown in NPC cells

**FIGURE 6:**
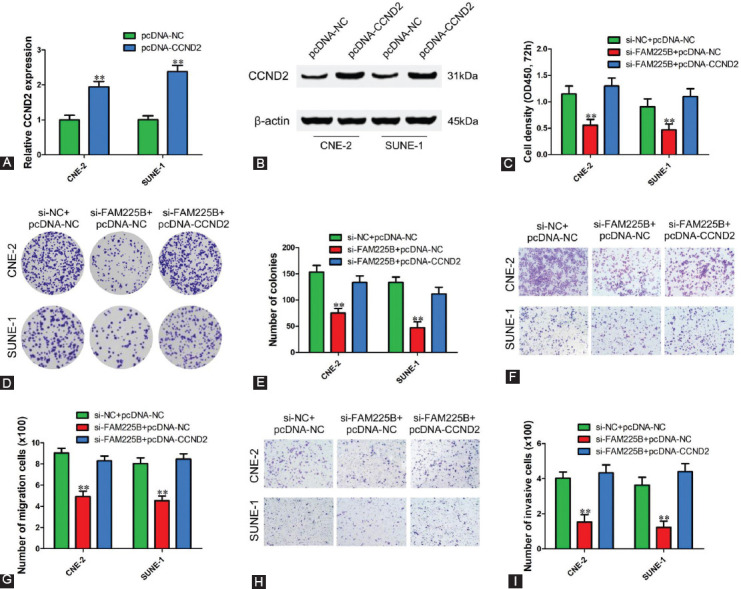
Overexpression of CCND2 reverses malignant phenotypes inhibition-induced by FAM225B knockdown in NPC cells. (A,B) qRT-PCR and Western blot were performed to validate the mRNA (A) and protein (B) level of CCND2 in CNE-2 and SUNE-1 cells transfected with pcDNA-NC or pcDNA-CCND2, respectively. (C) CCK-8 assay was used to determine the viability of CNE-2 and SUNE-1 cells after transfected with si-NC or si-FAM225B and pcDNA-NC or pcDNA-CCND2 for 72 h. (D,E) Colony formation assay was used to determine the proliferation of CNE-2 and SUNE-1 cells after transfected with si-NC or si-FAM225B and pcDNA-NC or pcDNA-CCND2. (F-I) Transwell assays with or without Matrigel were used to detect migration (F,G) and invasion (H,I) of CNE-2 and SUNE-1 cells transfected with si-NC or si-FAM225B and pcDNA-NC or pcDNA-CCND2. ** *p* < 0.01. CCND2: Cyclin D2; CCK-8: Cell counting kit-8; OD: Optical density; pcDNA-NC: pcDNA 3.1 negative control; pcDNA-CCND2: CCND2 overexpressing pcDNA 3.1 vector; si-FAM225B: Small interfering RNA targeting FAM225B; si-NC: Small interfering RNA negative control; qRT-PCR: Quantitative reverse transcription polymerase reaction.

To further determine whether FAM225B mediates malignant phenotypes of NPC cells through a CCND2-dependent manner, pcDNA-CCND2 or pcDNA-NC vector was co-transfected into CNE-2 and SUNE-1 cells with si-FAM225B or si-NC (Figure 6A and 6B). CCK-8 assay and colony formation assay results showed overexpression of CCND2 attenuated the inhibitory effect of FAM225B knockdown on cell proliferation (Figure 6C-6E). Transwell assay results showed that silence of FAM225B-mediated inhibition of NPC cell migration (Figure 6F and 6G) and invasion (Figure 6H and 6I) was significantly reversed by transfection of pcDNA-CCND2 compared with pcDNA-NC. These findings suggest that FAM225B promotes NPC growth and metastasis at least partly through regulating CCND2.

## DISCUSSION

Recently, a growing number of studies demonstrated that dysregulated lncRNAs played important roles in tumor growth, migration, invasion, stemness, and chemoresistance [24-26]. More importantly, they can be used as new prognostic markers and potential therapeutic targets for tumors [27-29]. In the current study, we reported that FAM225B was frequently upregulated in NPC tissues and cell lines. Our data also showed that high expression level of FAM225B was significantly associated with unfavorable overall survival of patients with NPC. We further analyzed the relationship between FAM225B expression and patients’ clinical feathers and found that upregulated FAM225B strongly correlated to TNM stage, T stage, and distant metastasis, which suggest that FAM225B might function as a useful prognostic biomarker and regulate NPC growth and metastasis.

To verify our hypothesis, loss-of-function experiments were performed *in vitro* and *in vivo*. The results showed that FAM225B knockdown prominently suppressed NPC cell proliferation, migration, and invasion. These results imply that FAM225B is an oncogene in NPC tumorigenesis, which is consistent with Lian’s report of FAM225B promoting bladder cancer cell invasion [13].

MicroRNAs (miRNAs) are a class of small noncoding RNAs that have vital roles as oncogenes or tumor suppressors during carcinogenesis [30-32]. miR-613, located on chromosome 12 at 12p13.1, has been found to be frequently down-regulated and act as tumor suppressor in many types of cancers, such as esophageal squamous cell carcinoma, ovarian cancer, hepatocellular carcinoma, osteosarcoma, and glioma [33-37]. In colon cancer and cervical cancer, however, miR-613 was up-regulated and functioned as oncomiRNA promoting cell proliferation and invasion [38,39]. Taken together, miR-613 has a complex and controversial function in different cancers, yet its role in NPC is still undefined.

Through bioinformatics analysis and luciferase reporter assay, miR-613 was identified as a candidate miRNA for FAM225B. We observed that the silence of FAM225B increased miR-613 expression in NPC cells. Conversely, restoration of miR-613 reduced FAM225B expression. These results indicate that there is an interactive regulation between FAM225B and miR-613. Furthermore, miR-613 in NPC tissues and cell lines was lower than that in adjacent normal tissues and NP69 cells. Our data also revealed that miR-613 level in NPC tissues was strongly associated with FAMM225B. Altogether, miR-613 might be an important downstream gene of FAM225B.

Similar to other lncRNAs, FAM22B might function as a competing endogenous RNA of miR-613 to influence mRNA or other lncRNAs by competitively binding to miRNA response elements. In this study, further investigations revealed that FAM225B functions as a ceRNA of miR-613 to upregulate CCND2 expression. CCND2 is a type of cell cycle regulatory gene that is abnormally expressed in a wide variety of tumors and involved in carcinogenesis and progression [18-23]. We found that CCDN2 was upregulated and high expression level of CCND2 predicted a poor prognosis of NPC patients. In addition, CCND2 overexpression was observed to reverse the suppression of FAM225B knockdown on proliferation, migration and invasion of NPC cells, indicating that FAM225B promotes cell proliferation and metastasis at least partly through regulating CCND2 expression.

## CONCLUSION

To sum up, our study demonstrated that FAM225B was significantly upregulated and predicted an unfavorable overall survival of NPC patients. Furthermore, FAM225B functioned as a ceRNA of miR-613 to increase CCND2 expression and promote NPC cell proliferation and metastasis. Altogether, our data highlights FAM225B as an oncogene to promote NPC progression and also adds new insights into the diagnosis and treatment of NPC.

## References

[1] Wei KR, Zheng RS, Zhang SW, Liang ZH, Li ZM, Chen WQ (2017). Nasopharyngeal carcinoma incidence and mortality in China, 2013. Chin J Cancer.

[2] Wei WI, Sham JS (2005). Nasopharyngeal carcinoma. Lancet.

[3] Sung H, Ferlay J, Siegel RL, Laversanne M, Soerjomataram I, Jemal A (2021). Global cancer statistics 2020:GLOBOCAN estimates of incidence and mortality worldwide for 36 cancers in 185 countries. CA Cancer J Clin.

[4] Kang Y, He W, Ren C, Qiao J, Guo Q, Hu J (2020). Advances in targeted therapy mainly based on signal pathways for nasopharyngeal carcinoma. Signal Transduct Target Ther.

[5] Wang S, Claret FX, Wu W (2019). MicroRNAs as therapeutic targets in nasopharyngeal carcinoma. Front Oncol.

[6] Pan JJ, Ng WT, Zong JF, Chan LL, O'Sullivan B, Lin SJ (2016). Proposal for the 8^th^ edition of the AJCC/UICC staging system for nasopharyngeal cancer in the era of intensity-modulated radiotherapy. Cancer.

[7] Chen MY, Jiang R, Guo L, Zou X, Liu Q, Sun R (2013). Locoregional radiotherapy in patients with distant metastases of nasopharyngeal carcinoma at diagnosis. Chin J Cancer.

[8] Wen X, Liu X, Mao YP, Yang XJ, Wang YQ, Zhang PP (2018). Long non-coding RNA DANCR stabilizes HIF-1a and promotes metastasis by interacting with NF90/NF45 complex in nasopharyngeal carcinoma. Theranostics.

[9] Zheng YJ, Zhao JY, Liang TS, Wang P, Wang J, Yang DK (2019). Long noncoding RNA SMAD5-AS1 acts as a microRNA-106a-5p sponge to promote epithelial mesenchymal transition in nasopharyngeal carcinoma. FASEB J.

[10] Hu X, Liu W, Jiang X, Wang B, Li L, Wang J (2019). Long noncoding RNA LINC00460 aggravates invasion and metastasis by targeting miR-30a-3p/Rap1A in nasopharyngeal carcinoma. Hum Cell.

[11] Wang X, Jin Q, Wang X, Chen W, Cai Z (2019). LncRNA ZFAS1 promotes proliferation and migration and inhibits apoptosis in nasopharyngeal carcinoma via the PI3K/AKT pathway *in vitro*. Cancer Biomark.

[12] Chen B, Wang C, Zhang J, Zhou Y, Hu W, Guo T (2018). New insights into long noncoding RNAs and pseudogenes in prognosis of renal cell carcinoma. Cancer Cell Int.

[13] Lian P, Wang Q, Zhao Y, Chen C, Sun X, Li H (2019). An eight-long non-coding RNA signature as a candidate prognostic biomarker for bladder cancer. Aging (Albany NY).

[14] Chen F, Li Z, Deng C, Yan H (2019). Integrated analysis identifying new lncRNA markers revealed in ceRNA network for tumor recurrence in papillary thyroid carcinoma and build of nomogram. J Cell Biochem.

[15] Li J, Zhang Q, Ge P, Zeng C, Lin F, Wang W (2020). FAM225B Is a Prognostic lncRNA for Patients with Recurrent Glioblastoma. Dis Markers.

[16] Ma X, Liu L (2021). Knockdown of FAM225B inhibits the progression of the hypertrophic scar following glaucoma surgery by inhibiting autophagy. Mol Med Rep.

[17] Ortega S, Malumbres M, Barbacid M (2002). Cyclin D-dependent kinases, INK4 inhibitors and cancer. Biochim Biophys Acta.

[18] Wang M, Wang Z, Zhu X, Guan S, Liu Z (2019). LncRNA KCNQ1OT1 acting as a ceRNA for miR-4458 enhances osteosarcoma progression by regulating CCND2 expression. *In Vitro* Cell Dev Biol Anim.

[19] Shen F, Chang H, Gao G, Zhang B, Li X, Jin B (2019). Long noncoding RNA FOXD2-AS1 promotes glioma malignancy and tumorigenesis via targeting miR-185-5p/CCND2 axis. J Cell Biochem.

[20] Hu W, Liu Q, Pan J, Sui Z (2018). MiR-373-3p enhances the chemosensitivity of gemcitabine through cell cycle pathway by targeting CCND2 in pancreatic carcinoma cells. Biomed Pharmacother.

[21] Jin M, Ren J, Luo M, You Z, Fang Y, Han Y (2020). Long non-coding RNA JPX correlates with poor prognosis and tumor progression in non-small-cell lung cancer by interacting with miR-145-5p and CCND2. Carcinogenesis.

[22] Wang S, Li X, Li ZG, Lu J, Fang WY, Ding YQ (2011). Gene expression profile changes and possible molecular subtypes in differentiated-type nonkeratinizing nasopharyngeal carcinoma. Int J Cancer.

[23] Li X, Liu F, Lin B, Luo H, Liu M, Wu J (2017). miR-150 inhibits proliferation and tumorigenicity via retarding G1/S phase transition in nasopharyngeal carcinoma. Int J Oncol.

[24] Wu K, Jiang Y, Zhou W, Zhang B, Li Y, Xie F (2020). Long noncoding RNA RC3H2 facilitates cell proliferation and invasion by targeting MicroRNA-101-3p/EZH2 axis in OSCC. Mol Ther Nucleic Acids.

[25] Zhao W, Geng D, Li S, Chen Z, Sun M (2018). LncRNA HOTAIR influences cell growth, migration, invasion, and apoptosis via the miR-20a-5p/HMGA2 axis in breast cancer. Cancer Med.

[26] Ren J, Ding L, Zhang D, Shi G, Xu Q, Shen S (2018). Carcinoma-associated fibroblasts promote the stemness and chemoresistance of colorectal cancer by transferring exosomal lncRNA H19. Theranostics.

[27] Su X, Zhang J, Yang W, Liu Y, Liu Y, Shan Z (2020). Identification of the prognosis-related lncRNAs and genes in gastric cancer. Front Genet.

[28] Shen X, Xue Y, Cong H, Wang X, Fan Z, Cui X (2020). Circulating lncRNA DANCR as a potential auxillary biomarker for the diagnosis and prognostic prediction of colorectal cancer. Biosci Rep.

[29] Zhang Y, Ma L, Wang C, Wang L, Guo Y, Wang G (2020). Long noncoding RNA LINC00461 induced osteoarthritis progression by inhibiting miR-30a-5p. Aging (Albany NY).

[30] Jansson MD, Lund AH (2012). MicroRNA and cancer. Mol Oncol.

[31] Calin GA, Croce CM (2006). MicroRNA signatures in human cancers. Nat Rev Cancer.

[32] Lin S, Gregory RI (2015). MicroRNA biogenesis pathways in cancer. Nat Rev Cancer.

[33] Guan S, Wang C, Chen X, Liu B, Tan B, Liu F (2016). MiR-613:A novel diagnostic and prognostic biomarker for patients with esophageal squamous cell carcinoma. Tumour Biol.

[34] Fu X, Cui Y, Yang S, Xu Y, Zhang Z (2016). MicroRNA-613 inhibited ovarian cancer cell proliferation and invasion by regulating KRAS. Tumour Biol.

[35] Wang W, Zhang H, Wang L, Zhang S, Tang M (2016). miR-613 inhibits the growth and invasiveness of human hepatocellular carcinoma via targeting DCLK1. Biochem Biophys Res Commun.

[36] Li X, Sun X, Wu J, Li Z (2016). MicroRNA-613 suppresses proliferation, migration and invasion of osteosarcoma by targeting c-MET. Am J Cancer Res.

[37] Sang Q, Liu X, Sun D (2018). Role of miR-613 as a tumor suppressor in glioma cells by targeting SOX9. Onco Targets Ther.

[38] Yang X, Zhang L, Song X, He W, Zhang D, Lu Q (2018). MicroRNA-613 promotes colon cancer cell proliferation, invasion and migration by targeting ATOH1. Biochem Biophys Res Commun.

[39] Li WT, Wang BL, Yang CS, Lang BC, Lin YZ (2018). MiR-613 promotes cell proliferation and invasion in cervical cancer via targeting PTPN9. Eur Rev Med Pharmacol Sci.

